# Genome Fragmentation Is Not Confined to the Peridinin Plastid in Dinoflagellates

**DOI:** 10.1371/journal.pone.0038809

**Published:** 2012-06-18

**Authors:** Mari Espelund, Marianne A. Minge, Tove M. Gabrielsen, Alexander J. Nederbragt, Kamran Shalchian-Tabrizi, Christian Otis, Monique Turmel, Claude Lemieux, Kjetill S. Jakobsen

**Affiliations:** 1 Department of Biology, Centre of Ecological and Evolutionary Synthesis (CEES), University of Oslo, Oslo, Norway; 2 Department of Biology, Microbial Evolution Research Group (MERG), University of Oslo, Oslo, Norway; 3 Département de biochimie, de microbiologie et de bio-informatique, Université Laval, Québec, Canada; American University in Cairo, Egypt

## Abstract

When plastids are transferred between eukaryote lineages through series of endosymbiosis, their environment changes dramatically. Comparison of dinoflagellate plastids that originated from different algal groups has revealed convergent evolution, suggesting that the host environment mainly influences the evolution of the newly acquired organelle. Recently the genome from the anomalously pigmented dinoflagellate *Karlodinium veneficum* plastid was uncovered as a conventional chromosome. To determine if this haptophyte-derived plastid contains additional chromosomal fragments that resemble the mini-circles of the peridin-containing plastids, we have investigated its genome by in-depth sequencing using 454 pyrosequencing technology, PCR and clone library analysis. Sequence analyses show several genes with significantly higher copy numbers than present in the chromosome. These genes are most likely extrachromosomal fragments, and the ones with highest copy numbers include genes encoding the chaperone DnaK(Hsp70), the rubisco large subunit (rbcL), and two tRNAs (trnE and trnM). In addition, some photosystem genes such as psaB, psaA, psbB and psbD are overrepresented. Most of the dnaK and rbcL sequences are found as shortened or fragmented gene sequences, typically missing the 3′-terminal portion. Both dnaK and rbcL are associated with a common sequence element consisting of about 120 bp of highly conserved AT-rich sequence followed by a trnE gene, possibly serving as a control region. Decatenation assays and Southern blot analysis indicate that the extrachromosomal plastid sequences do not have the same organization or lengths as the minicircles of the peridinin dinoflagellates. The fragmentation of the haptophyte-derived plastid genome *K. veneficum* suggests that it is likely a sign of a host-driven process shaping the plastid genomes of dinoflagellates.

## Introduction

The dinoflagellates are a diverse group of protists comprising both heterotrophic and phototrophic lineages. Most of the phototrophic species harbor a plastid of red algal origin characterized by the carotenoid peridinin, chlorophyll a and c_2_ [1], [2]. The peridinin-containing plastid is most likely the ancestral plastid in dinoflagellates. As a result of a massive loss of plastid genes from the endosymbiont, only 18 different genes have been identified in total among all investigated genomes. These are found on minicircles [3] of typically 2–3 kb length rather than on a single chromosome. On the other hand, some dinoflagellate species have differently pigmented plastids that have evolved through replacement of the ancestral plastid either by endosymbiosis of an alga with a primary plastid or by an alga with a secondary plastid [1], [4], [5], [6]. Algae that gave rise to plastid replacements (serial endosymbiosis; see [7] for a review) in dinoflagellates include cryptophytes, haptophytes, diatoms and green algae [8], [9], [10], [11], [12], [13], [14], [15], [16], [17]. It is currently not known whether serial endosymbiosis in dinoflagellates in one way or another also involves desintegration of the endosymbiont genome into minicircles as seen in the peridinin plastids.

Among dinoflagellates that have been involved in serial transfer of plastids, we find the family Kareniaceae, consisting of *Karenia*, *Karlodinium* and *Takayama* [18], harboring haptophyte-derived plastids with fucoxanthins as accessory pigments [2], [4], [6], [19]. The fucoxanthin-plastid of *Karlodinium veneficum* is well integrated into the host [2], [20]. Recently, the plastid genome of *K.veneficum* was sequenced and demonstrated to be organized conventionally as a genome of minimum 143 kb [21]. However, the genome has undergone extensive gene loss and expanded in size compared to its homolog in the haptophyte *Emiliania huxleyi*. Particularly, ancestral gene clusters are broken up, and intergenic regions are extended [21]. Several genes exhibit unusual features such as lack of conserved regions and reading frames distorted by stop codons or frame shifts.

In the present study, we have examined whether the *Karlodinium* plastid genome has undergone fragmentation similar to what is known for the peridinin-containing plastid. We have investigated the *K.veneficum* plastid genome by in-depth sequencing using 454 pyrosequencing technology, PCR and clone library analyses. The data indeed reveal massive variation of copy number and many variants of the genes present in the plastid. Most likely, these genes are extrachromosomal fragments organized in complex structures, revealing that genome reduction, re-arrangements and fractionation among dinoflagellates plastids have taken place independently in at least two different types of plastid lineages. This suggests that the evolutionary process is host-related and a characteristic feature of the dinoflagellate host.

## Results

### High Throughput Sequencing Shows High Copy Numbers of Certain Plastid Genes

**Figure 1:**
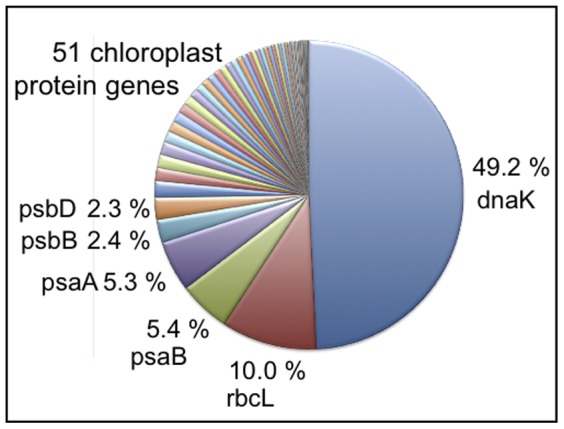
Result of a tblastn search using 119 plastid proteins from *Emiliania huxleyi* against the *K. veneficum* 454 GS FLX data set. The E-value cut-off was 10^−5^.

The data set generated by 454 pyrosequencing of plastid enriched DNA (see methods) was used to analyze gene abundance and gene variability in *K. veneficum*. Since the plastid genome is of haptophyte origin, blast searches using the haptophyte *Emiliania huxleyi* protein encoding genes against *K. veneficum* pyrosequencing reads were performed. The results displayed in Figure 1 show that as much as 49% of the hits were dnaK, and 10% were rbcL. Together with 4 photosystem-associated genes (psaA, psaB, psbB, and psbD), dnaK and rbcL comprised 75% of the sequences analyzed. Sequence analysis of clones containing dnaK and rbcL revealed that these genes were to a large extent incomplete. Analysis of the *K. veneficum* plastid clone library showed a similar result (not shown).

### The rbcL Gene Sequences Occur in Variants of Different Lengths

Since some genes were found in high copy numbers, we proceeded by testing for the presence of minicircles in *K. veneficum*. Using outwards-directed primers specific for dnaK and rbcL (see Table S1), PCR yielded strong bands on agarose gels. The PCR products were cloned and sequenced, revealing fragmented or incomplete gene sequences for both genes.

**Figure 2:**
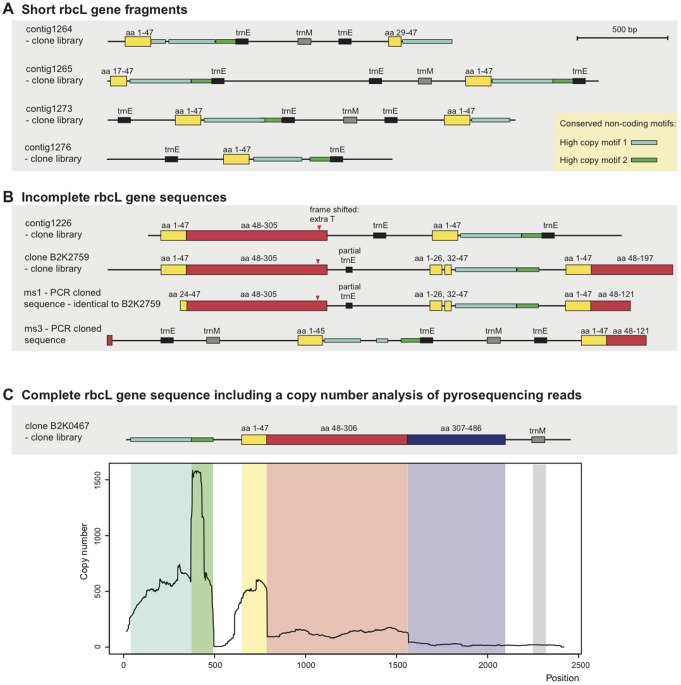
Schematic overview of rbcL sequences found in *K.veneficum*. Sequences are either library clones or PCR-clones generated with outwards directed primers. A. Sequences containing a short rbcL gene fragment, corresponding to the first 47 amino acids. B. Sequences containing the incomplete rbcL gene sequences, corresponding to amino acids 1–306. C. Sequence containing the complete rbcL gene. A copy number analysis of pyrosequencing reads mapping to the rbcL gene is displayed below. The analysis shows number of read occurences per base of the cloned rbcL sequence.

Figure 2 presents all the rbcL sequences derived from the PCR clones, the clone library and the pyrosequencing data, revealing that the sequences fall into three groups based on structural organization. The first group of rbcL sequences (A) contains only the coding region for the first 47 amino acids, followed by conserved non-coding sequence motifs and tRNA genes (trnE and trnM). The second group (B) contains both longer coding regions of the rbcL protein, up to amino acid 304–306, and the same short rbcL version as in A). All clones in B) have a frame shift due to an extra T between the base triplets encoding amino acids 292 and 293, indicating that RNA editing is needed for correct expression of the gene.

A complete rbcL sequence coding for 486 amino acids, without frameshifts, was found in the clone library (C). Noticeably, the tRNA genes trnE(uuc) and trnM(cau) are found interspersed with rbcL; the PCR cloned sequence ms3 contains as many as three trnE genes and two trnM genes.

### Parts of the rbcL Locus are Highly Overrepresented in the Pyrosequencing Data

A coverage analysis giving the number of occurrences per base in the assembled rbcL locus is presented in Figure 2C. The number of pyrosequencing reads found for the different parts of the rbcL gene corresponds remarkably well with the library and PCR clones that were shown in Figure 2A–C. The first part of the rbcL gene is present at high copy numbers, whereas the middle part of the gene sequence has intermediate occurrence. The end of the gene, which is missing in most clones, has low occurrence in the plastid data set. Two motifs that are found upstream of the gene, High copy motif 1 and High copy motif 2, show the highest peak of sequence occurrence. These elements were recognized as common elements among all rbcL clones.

### Southern Blot Analysis Confirms the Coverage Analysis of the rbcL Locus

**Figure 3:**
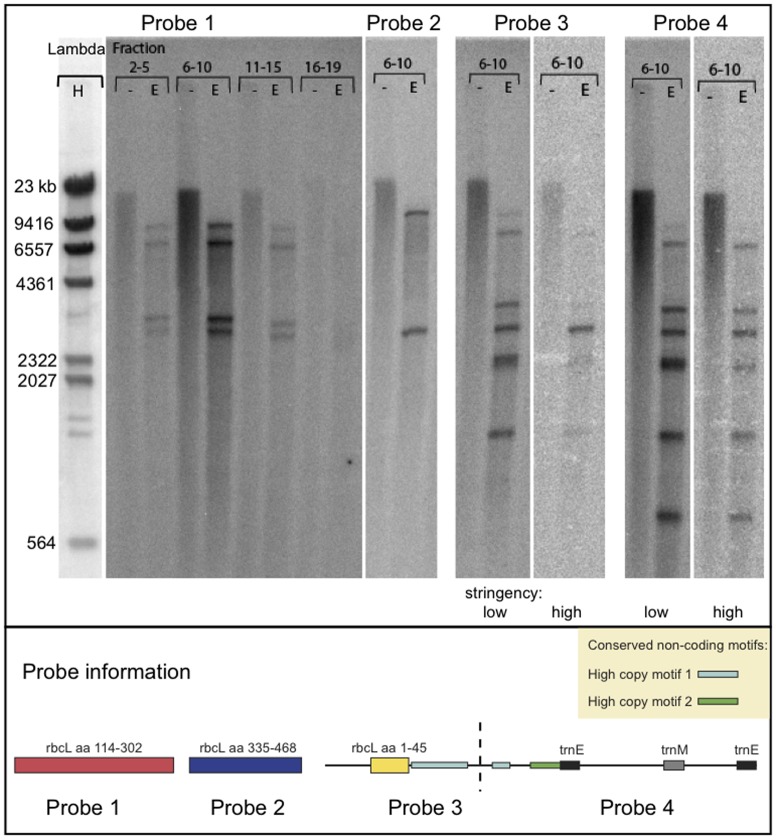
Southern blot analysis of rbcL. Two filters containing pooled CsCl fractions of *K. veneficum* DNA, either undigested (−) or digested (E) with EcoRI, were prepared. Probe 1 and 2 contain rbcL coding sequence, whereas probe 3 and 4 are derived from the PCR cloned sequence ms3 displayed in Figure 2B. Filter I was first hybridized with probe 1, stripped, and rehybridized with probe 4. Filter II was hybridized with probe 2, stripped and rehybridized with probe 3 (filter II). Filter I was finally hybridized with the Lambda/HindIII marker shown to the left in the figure.

We hybridized different parts of the rbcL sequences to a Southern blot containing pooled fractions of uncut and EcoRI-digested DNA from the CsCl gradient. The hybridization patterns shown in Figure 3, which were obtained with the rbcL probes, are consistent with the gene structures found in Figure 2. The first probe, corresponding to the middle part of the rbcL gene (probe 1), hybridizes to four bands. The second probe, the end part of rbcL missing in most clones (probe 2), only hybridizes to two bands. The third probe, containing the 5′ coding region of rbcL hybridizes to at least seven bands, and the fourth probe, with common non-coding elements, hybridizes to at least eight bands. At high stringency conditions (at 70°C washing temperature), only one target band is detected with the 5′ coding probe. Probe 4, containing putative common regulatory elements, hybridizes equally strong to all bands, indicating that this probe sequence has regulatory elements common to several gene targets.

Notably, a high molecular weight DNA smear is observed in the lanes containing uncut DNA, showing that the hybridization targets are distributed over a large size range rather than forming a distinct band.

### K. Veneficum Contains Shortened and Fragmented dnaK Gene Sequences

**Figure 4:**
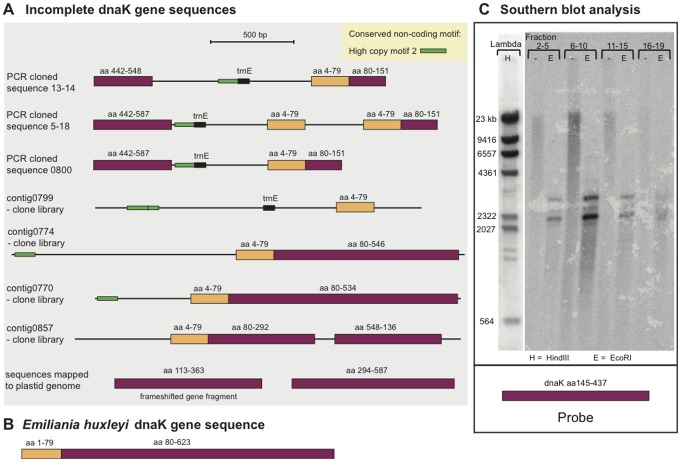
Schematic overview of dnaK sequences found in *K.veneficum*. PCR-clones generated with outwards directed primers; library clone contigs; and plastid genome contigs assembled from pyrosequencing reads. A. Partial dnaK sequences found in *K.veneficum*. B. The complete gene (aa 623) from *E. huxleyi*. C. Southern blot analysis of dnaK. Hybridization was performed with filter II (see Figure 3). The filter II was hybridized with a radiolabelled dnaK probe generated by PCR, using inwards-directed primers. The probe contains the coding sequence for amino acids 145–437.

A diverse collection of library clones and PCR cloned sequences (see methods) is presented in Figure 4A, including two partial dnaK genes which could be mapped to the conventional plastid genome [21]. The dnaK versions are either fragmented or truncated. The dnaK fragments are of the plastid version of the gene; i.e. related to the cyanobacterial dnaK gene. PCR amplification using dnaK-specific outwards-directed primers produced abundant products of 1800–2000 bp length. We were able to clone products of length 1489 bp, 1741 bp and 2059 bp, respectively (PCRout clones of Figure 4A). Inwards directed primers generated a product of 882 bp. If these sequences are part of circular molecules or repeat units in the case of tandem arrangements, the cloned sequences mentioned above would correspond to units of ca. 2300, 2600 and 2900 bp in size. The coding sequences end at amino acid position 548 or 587, compared to 623 amino acids in the *E. huxleyi* plastid (Figure 4B). Similar to the short rbcL sequences, there are separate occurrences of the 5′ coding region of dnaK (corresponding to aa 4–79 in *E. huxleyi*). One clone differs from another only by lacking the separate occurring 5′ coding region; the sequences are otherwise identical, suggesting that one sequence is derived from the other.

Southern blot hybridization with dnaK coding sequence shows two strongly hybridizing bands of approx. size 2300 and 3000 bp, probably corresponding to the cloned putative minicircles or putative repeat units, each sequence containing one site for the restriction enzyme EcoRI (Figure 4C). One of the conserved upstream motifs identified in rbcL sequence variants, the highly abundant motif 2, is recognized upstream of most dnaK variants. The situation for dnaK resembles that of rbcL, suggesting an organization of these genes in high copy numbers outside of the conventional plastid genome.

### Occurrence of a Common Upstream Motif in rbcL and dnaK Gene Sequences

A closer look at the pyrosequencing reads sharing the ca. 120 bp long conserved motif 2 shows that the sequence is highly conserved, depicted in graphical form as a Sequence Logo (Figure S1A). We find that just a handful of 895 analyzed sequences corresponds to the conventional plastid genome of *K. veneficum*. The conventional genome contains a complete rbcL gene in this position. However, most motif 2 sequences display a downstream trnE gene, followed by sequence divergence into different groups. We identified two of the groups as non-coding rbcL and dnaK sequences. Within each group of sequences, we observe numerous point mutations and evidence of slipped strand mispairing. The motif forms elaborate secondary structures (not shown). By sorting the 454-generated sequences in forward and reverse reads it can be shown that around 95% of the forward reads terminate at the start of trnE (Figure S1B), whereas the reverse reads traverse this sequence. The PCR polymerase used for 454 sequencing apparently responds to trnE as a termination signal in the *in vitro* situation.

Since one of the two pyrosequencing experiments was performed on DNA that was first amplified by RCA, we also examined sequences generated by direct PCR, without the use of RCA. A sequence alignment of the conserved element is shown in Figure S2. Notably, this data set does not differ from the 454 pyrosequencing data. The figure also includes Sanger sequenced library clones, where RCA was used in library construction. We are well aware that a data set generated by using RCA prior to sequencing could potentially contain artifacts of such character. We are therefore reassured by finding the same type of sequence variation in data generated without the use of RCA and in Sanger sequences from the clone library and from the PCR-out clones.

### Concatenated DNA Circles not Found

**Figure 5:**
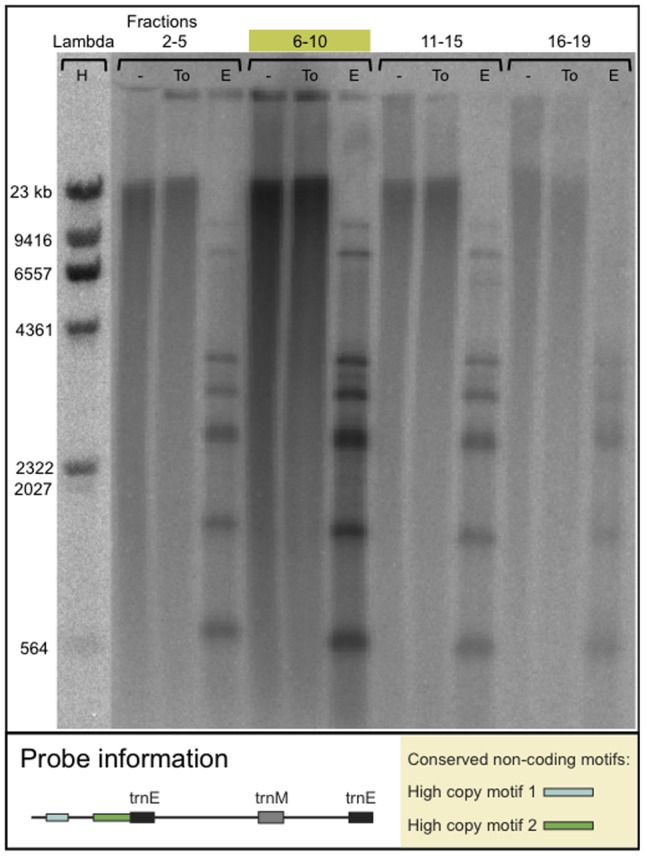
Southern blot analysis of DNA treated with Topoisomerase IV. The filter contains pooled CsCl fractions of *K. veneficum* DNA, undigested (−), treated with Topoisomerase IV (To), and digested with EcoRI (E). The hybridization probe is identical to probe 4 of Figure 3, containing the highly conserved motif 2 and a partial sequence of motif 1. The location of the plastid genome is indicated by green shade above the lanes.

**Figure 6:**
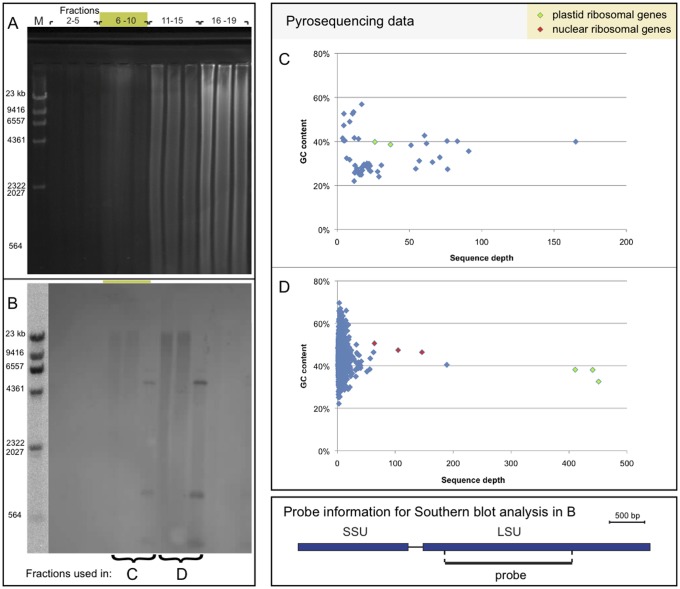
LSU hybridization and ribosomal gene coverage. The location of the plastid genome is indicated by green shade above the lanes. A. Agarose gel corresponding to blot in panel B. B. Southern blot analysis using a plastid LSU probe (same filter as in Figure 5). C. Read depth of pyrosequenced data from plastid-enriched DNA, pooled fractions 6–10 (corresponding to the FLX data set), plotted against GC content. D. Read depth of pyrosequenced data from pooled fractions 11–14, enriched in LSU sequences (Titanium data set) plotted against GC content. Colour code for pyrosequenced reads: Green, plastid ribosomal genes; red, nuclear ribosomal genes; blue, all other reads.

The absence of minicircle bands on Southern blots with undigested DNA may potentially be caused by minicircles organized in complex structures. Using a Topoisomerase of type II (*E.coli* DNA topoisomerase IV) on CsCl-fractionated DNA, we tested if the extrachromosomal genes exist as concatenated gene minicircles in *K.veneficum* plastid DNA. The enzyme is capable of decatenating linked circular molecules by producing transient double strand breaks [26]. We could not detect any bands using the non-coding rbcL probe with the putative regulatory element (Figure 5) or a plastid LSU probe (Figure 6B). Positive controls for the assay included successful decatenation of kDNA and relaxation of a supercoiled plasmid. These results suggest that higher order DNA structures similar to the interlocked DNA circles of trypanosomatid protozoa [27] do not exist in *K. veneficum*.

### Organization of Plastid Ribosomal Genes

The plastid LSU rRNA gene probe was hybridized to a Southern blot containing pooled fractions of the CsCl gradient DNA. An agarose gel image of the DNA is shown in Figure 6A. In this gradient, the plastid-enriched DNA is mainly located in the pooled fractions 6–10, whereas the nuclear DNA is found in the denser fractions 16–19. The plastid LSU probe somewhat unexpectedly hybridized to fractions 11–15; i.e. close to the nuclear DNA fractions (Figure 6B). In Figures 6C and 6D, copy numbers of pyrosequencing reads are plotted against GC content for the fractions 6–10 and 11–14. The plastid ribosomal genes are highly overrepresented in fractions 11–14, co-located with nuclear ribosomal genes and mitochondrial sequences (Table S3). The overall GC content of the conventional plastid genome is 27.1%, but the part containing the ribosomal genes has 38.1% GC. A GC plot of the entire plastid genome is shown in Figure S3.

The presence of the majority of plastid ribosomal DNA in denser fractions than the plastid genome, suggests that the ribosomal genes may additionally occur in different genomic contexts such as nuclear DNA or as an extrachromosomal fraction. One unit of the ribosomal genes consists of the genes rrf-rrs-rrl, and the copy number of this unit in the plastid genome of *K. veneficum* has not been exactly determined [21]. Using the RunViewer program to connect contigs, only tandem copies of the rDNA unit were found in the data set from the chloroplast-enriched fractions, whereas in the denser fraction (richer in ribosomal genes), head-head and tail-tail connections were mostly found (data not shown). Our data demonstrate the existence of at least two different versions of a sequence connecting units of the complete ribosomal genes in direct repeats, as shown in Figure S4. One sequence variant, less abundant than the published sequence [21], carry an 18 bp deletion in the junction between the units of the ribosomal genes. Hence, the ribosomal genes occur in multiple copies most likely in different genomic locations.

## Discussion

### The K. Veneficum Contains a Conventional Plastid Genome and a Satellite Fraction of Fragmented Genes

The *K.veneficum* plastid genome has previously been shown to exist as a conventional genome comprising genes of haptophyte origin [21]. The finding of high copy numbers of certain sequences combined with a remarkably high diversity of gene variants, lead us to conclude that parts of the plastid genome most likely exist in a fragmented form outside the conventional plastid genome. This applies to fragmented or shortened versions of rbcL and dnaK, with interspersed trnE and trnM genes. The photosystem genes psaA, psaB, psbB and psbD, shown to be on minicircles in peridinin dinoflagellates [3], [28], are also found in high copy numbers in the 454 pyrosequencing data. Interestingly, the photosystem gene psbA, as well as other related genes to rbcL and dnaK such as rbcS and groEL were not found among the amplified genes. rbcS and groEL are shown to be present within the conventional plastid genome [21]. The plastid ribosomal genes were also mapped to the conventional plastid genome [21]. By analyzing pyrosequencing reads derived from CsCl gradient fractions of different densities, we were able to show that the bulk of the plastid LSU rRNA gene was separated from the conventional plastid genome. In dinoflagellate nuclear genomes, many genes occur as tandem repeats [29] or sometimes as clusters of tandem repeats [30], [31]. If the plastid rRNA operon of ca. 6 kb per unit occurred in tandem repeats, a number of units in the order of magnitude 10 tandem repeats would explain the separation we observed on the density gradient. Alternatively, the repeating units of rRNA genes could be located in the nuclear genome. As far as we know, none of these possibilities have been observed before. A further option would be that the plastid rDNA is present extrachromosomally in the organelle, similar to the situation in peridinin dinoflagellates. The most likely scenario here would be repeats on larger circles. Such circles could even be localized in the nucleus, as has been shown for plastid-derived single gene minicircles in the dinoflagellate *Ceratium horridum* [32].

All genes found in high copy numbers are of haptophyte origin, indicating that the process of transferring genes to extrachromosomal fragments seen in peridinin-plastid dinoflagellates, has also occurred in *K.veneficum.* The extrachromosomal sequences appear to have variable sizes, and the genes rbcL and dnaK occurring in highest copy numbers in *K.veneficum* are not among the minicircle genes of peridinin dinoflagellates. The high molecular weight smear observed with uncut DNA on Southern blots, was probably not due to higher order structures of interlocked circles, as shown by the topoisomerase experiment. The peridinin minicircles of *Heterocapsa triquetra* did not contain catenated minicircles either [33]; hence these structures are probably absent among the dinoflagellates. The plastid DNA structures found in *H. triquetra* were rather shown to contain rolling circle replication (RCR) intermediates [33], suggesting that the minicircles of peridinin dinoflagellates replicate via a RCR mechanism. The intermediates had variable form and size, as observed by imaging studies and Southern blot analysis. One possible scenario explaining our Southern blot results with rbcL and dnaK, not showing hybridization for low molecular bands, would be an organization in tandem repeats in the conventional plastid genome. On the other hand, the large number of rbcL and dnaK variants, does not support this alternative. As argued for the location of the ribosomal genes, a possible organization of the fragmented and highly variable plastid genes would be a location on larger extrachromosomal DNA structures, but this has to be investigated further.

### Plastid Localization of the Amplified Genes

If the amplified plastid genes were localized in the nuclear DNA of *K. veneficum,* plastid targeting signals would be expected to be present. We were unable to find any evidence for such targeting sequences. The amplified plastid genes are found in the same fractions (in the CsCl gradient) as the conventional plastid genome, and these sequences are highly AT-rich, unlike nuclear DNA. It should be emphasized that our data do not definitely prove that the high copy number plastid genes are localized to the photosynthetic organelle, but given the current data we strongly favor this scenario.

### Essential Plastid Genes Present in High Copy Numbers

The genes occurring in high copy numbers of highly variable sequence in *K. veneficum*, are among the plastid genes being retained in non-photosynthetic organisms. [34]. It has previously been shown that the gene encoding a subunit of the enzyme responsible for the first major step of carbon fixation in photosynthesis, rbcL, is essential in non-photosynthetic organisms [35]. The dnaK gene has also been shown to be retained in plastids that have become non-photosynthetic [36]. Both gene products are used in connection with oxidative stress; the N-terminal end of rbcL, present in the short versions of rbcL identified in *K.veneficum*, has been shown to have an additional function in binding to RNA [37], [38], and dnaK contributes in refolding of proteins [36], [39]. The two tRNAs encoded by trnE and trnMf, tRNA^Glu^ and the initiator tRNA^Met^, are predicted to be retained in plastid genomes of non-photosynthetic organisms according to the ‘essential tRNA hypothesis’ [34]: tRNA^Glu^ is needed as precursor in the biosynthesis of haem, chlorophyll and other tetrapyrroles in plants, algae and most bacteria [40], while tRNA^Met^ plays a role as initiator tRNA in organellar protein synthesis, as formylated methionyl-tRNA [34], [41]. The main part of the *K. veneficum* plastid genome contains a full set of tRNA genes, except for trnMf [21]. A possible advantage of organizing the essential genes outside of a conventional plastid genome is that the copy number can be used as a part of the gene regulation. This is supported by a study of A*mphidinium operculatum*, in which minicircle copy numbers were shown to be regulated in response to growth conditions [42]. Here, we have shown that the trnE gene is likely to function as a termination signal (see Fig. S1), and propose that the conserved element may be a control region for replication of extrachromosomal plastid gene fragments.

### Evolutionary Implications

A common feature of the apicomplexan and dinoflagellate lineages is the reduction of plastid gene numbers [28], [41]. The *K.veneficum* plastid genome has lost more than one third of the genes found in the haptophyte donor [21], indicating massive loss of genes throughout the integration of the plastid to the new host. A similar massive gene loss is also observed for the alveolate *Chromera velia*, a photosynthetic sister lineage to parasitic apicomplexa. *C. velia* has retained only 56 protein-coding genes in the plastid genome [43]. Notably, other dinoflagellate organelles have been subject to drastic changes. Dinoflagellate mitochondrial genomes have, unlike their apicomplexan homologs, their genes scattered on many small chromosomes [44], [45]. Intriguingly, the fragmentation process apparently happening in the haptophyte-derived plastid in *K. veneficum* resembles this fragmentation of dinoflagellate mitochondrial genes. The haptophyte origin of the genes as well as the presence of dnaK and rbcL of type I – not found as minicircles in the peridinin plastid –indicate that the plastid genome fragmentation in *K. veneficum* has occurred independently of the fragmentation of the peridinin plastid genome. The host environment, however, seems to work in a convergent fashion on genes of both *Karlodinium* and peridinin plastid genomes. In *Heterocapsa triquetra* five closely related minicircles were characterized that share a common element, 9G-9A-9G, and contain fragments of four different genes [46]. Here, a model is proposed explaining the generation of the five minicircles from a common ancestor by means of recombination, deletions and duplications. In two species of *Alexandrium, A. tamarense* and *A. catanella*, several differentially rearranged versions of psbA and psbD are found in addition to the standard gene copies [47]. The rearrangements were comprised of insertion and deletion mutations, indicating that replication-based repeat- mediated recombination was responsible for generation of the variants [47]. Both cases resemble the variable gene versions found in *K. veneficum*. We find variants of rbcL and dnaK that differ only by insertions/deletions. The relationship between the rearranged gene sequences and the corresponding standard genes is unclear. The rearranged genes could be signs of a plastid genome breaking down, and consequently – as a final stage – could lead to loss of the plastid (and subsequently uptake of a new endosymbiont – i.e. serial transfer). Altogether, it seems that dinoflagellates have a unique ability to evolve unusual fragmented organelle genomes. The involvement of the host replication system in the process and the role that the fragmented plastid genomes are playing are key issues to address in future.

## Materials and Methods

### Generation of Karlodinium Veneficum Plastid Sequences

*Karlodinium veneficum,* strain UIO 083 was originally isolated by Karl Tangen from Oslofjorden, Norway. Culturing, plastid DNA isolation and sequencing have been described elsewhere [21]. In short, plastid DNA was isolated from total DNA by CsCl gradient separation. Plastid DNA was identified in collected fractions by Southern blot hybridization with rbcL, psaA and psbA gene probes. Pooled plastid-rich fractions (fractions 6–10 of 40 fractions) were amplified by rolling circle amplification (RCA) and used for generation of a clone library. Plastid-containing clones (9600 clones) were sequenced by Sanger sequencing. In addition, the RCA-amplified, plastid-rich fractions were sequenced at the 454 GS FLX platform at the Norwegian High-Throughput Sequencing Centre (NCS; www:sequencing.uio.no) at CEES, University of Oslo, generating approx. 250,000 reads of average length 195 bp. Since the large subunit (LSU) rRNA gene was shown to be mainly located to later gradient fractions, another round of pyrosequencing (using the Titanium upgrade) was performed on non-amplified DNA (fractions 11–14) at the 454 platform, generating ca. 625,000 sequences of average length 290 bp.

### Sequence Analysis

Assembly of the plastid contigs has been described in [21]. Analysis of sequences not mapped to the genome contigs was performed using Sequencher 4.9. Genes were identified by Blast searches (http://blast.ncbi.nlm.nih.gov/) and tRNA genes analyzed using the tRNAscan-SE 1.21search server (http://selab.janelia.org/tRNAscan-SE/). Sequence alignments were made using the Mafft program [22] and MacClade 4 [23].

### PCR Cloning of Putative Minicircles

Plastid DNA-rich fractions were used as template for PCR with outwards-directed primers for amplification of dnaK and rbcL to test for the presence of minicircles. For this purpose, we used the DNA polymerase enzymes Phusion DNA polymerase (Finnzymes, Finland) and BD Advantage (Clontech, CA, USA). Primer sequences are given in Table S1. Notably, the DNA used in PCR had not been subjected to RCA. PCR products for the genes dnaK, rbcL and psaA were cloned using the TOPO TA Cloning Kit (Invitrogen, Carlsbad, CA, USA) and sequenced by the Sanger method.

### Southern Blot Analysis

A pooled fraction of DNA from the CsCl gradient was applied either undigested or digested with EcoRI on an agarose gel. From one agarose gel run, duplicate blots were prepared and hybridized with fragment probes that are specific to the LSU rRNA gene or distinct regions of rbcL and dnaK [24], [25]. All fragments were generated by PCR (primer sequences are given in Table S2) using cloned sequences in pSMART-HCKan (Lucigen Corporation, Middleton, WI) or in pCR2.1-TOPO (Invitrogen Corporation, Carlsbad, CA) and were purified using the Wizard SV Gel and PCR Clean-Up System (Promega Corporation, Madison, WI) prior to labeling. The rbcL and dnaK fragments were labeled using the DecaLabel™ DNA Labeling Kit (Fermentas, Burlington, ON, Canada), whereas the 1844 bp LSU rDNA fragment was labeled and detected with the random primed labeling kit DIG-High Prime DNA Labeling and Detection Starter Kit I (Roche Diagnostics Corporation, Indianapolis, IN). The blots were prepared for the next hybridization by washing them with boiling 1 mM EDTA, pH 8.0. All hybridizations with plastid probes were performed at 63°C, whereas hybridization with the Lambda marker was carried out at 68°C. To increase the stringency condition (see Figure 4), the washing temperature was raised to 70°C for another 2 h of washing before a new exposure. Hybridization signals arising from radioactive probes were detected with the Typhoon Variable Mode Imager (GE Healthcare, Chalfont St. Giles, United Kingdom).

### Topoisomerase II Assay

To test whether the putative minicircles were entangled together in complex stuctures, we treated the *K. veneficum* DNA fractions with Topoisomerase IV (Topogen, Port Orange, FL, USA). Per 10 µl sample containing 3.75 µl of combined CsCl fractions in the kit assay buffer, 5 U Topoisomerase IV were added. The reactions were incubated at 37°C overnight and then stopped with 1% SDS and Proteinase K digestion (50 µg/ml, 15 min. at 56°C). kDNA was used as positive control for decatenation. The Southern blot was prepared and hybridized as described, but ethidium bromide was omitted in the agarose gel.

## Supporting Information

Figure S1 **The conserved upstream element Motif 2: 895 copies from pyrosequencing data (blasting with E value cut-off of 10^−25^).** A. Graphical representation (Sequence Logo, [48]) of 895 aligned sequences of the conserved Motif 2 identified in *K. veneficum* plastid sequences. B. 895 aligned DNA sequences containing Motif 2 and trnE in putative minicircles shown in MacClade (pretty print matrix). Left panel; forward and complementary reads aligned together. Right panel; forward reads and complementary reverse reads aligned in separate blocks.
(TIFF)

Figure S2 **Aligned sequences of Motif 2 in library clones.** The gene structures of the library clones are shown in Figures 2 and 4.
(TIFF)

Figure S3 **GCView** [**49**] **of the conventional plastid genome** [**21**] **of** ***Karlodinium veneficum*** **.**
(TIFF)

Figure S4 **Aligned sequence connecting units of plastid ribosomal genes.** The location the sequence in the plastid **genome** of *K. veneficum* is indicated above the sequences.
(TIFF)

Table S1 **PCR primers used for outwards directed amplification of putative minicircle genes.**
(DOCX)

Table S2 **PCR primers used for amplification of probes used in Southern blot hybridization.**
(DOCX)

Table S3 **Sequence coverage in 454 sequences generated from pooled CsCl fractions 11–14.** The coverage is calculated as the read depth for each base, averaged over the contig.
(DOCX)
