# Supplementary figures and images for: Genome Fragmentation Is Not Confined to the Peridinin Plastid in Dinoflagellates
Source: PLoS One. 2012 Jun 18;7(6):e38809. doi: 10.1371/journal.pone.0038809 (PMC3377699; doi:10.1371/journal.pone.0038809)

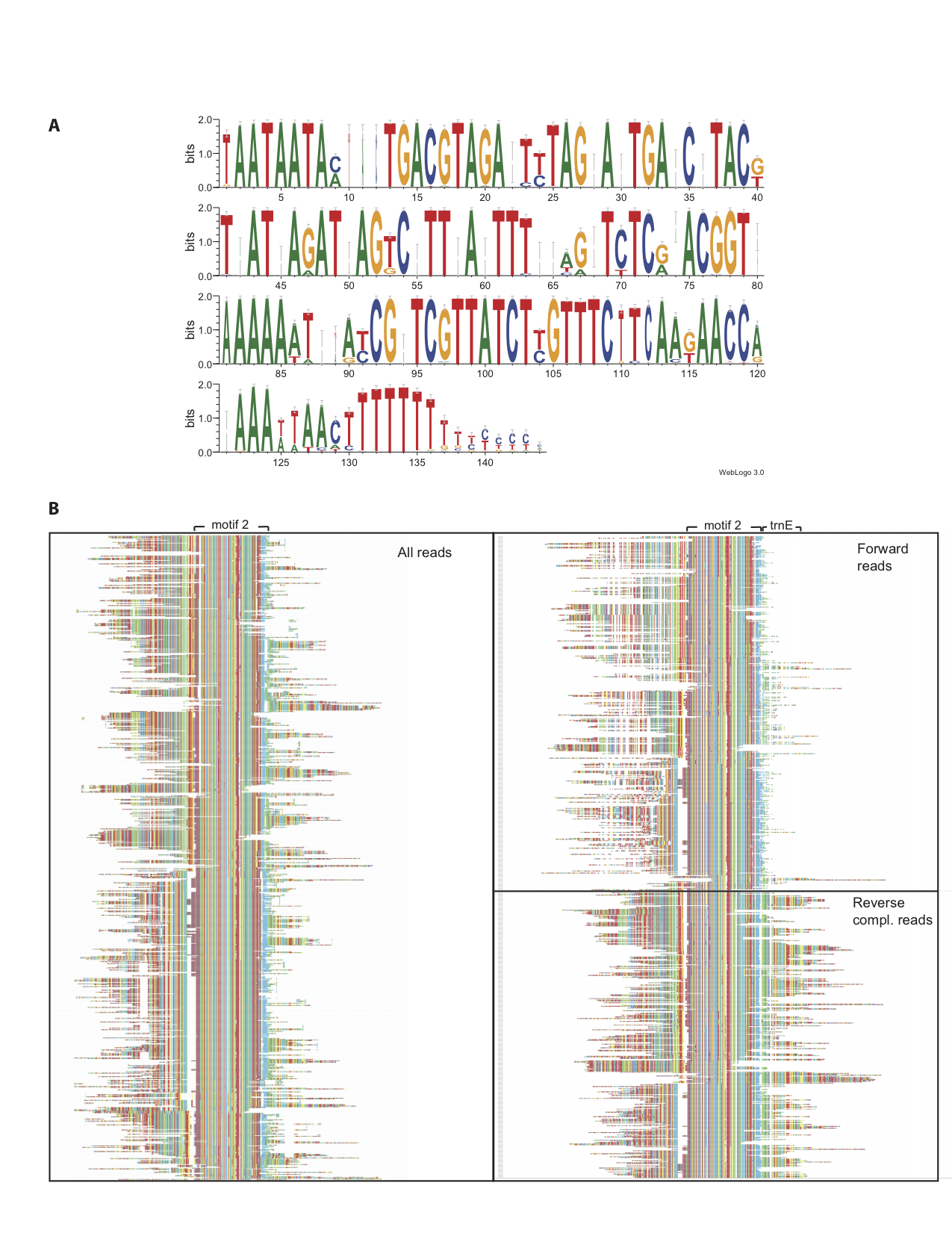

Supplement: Figure S1 — The conserved upstream element Motif 2: 895 copies from pyrosequencing data (blasting with E value cut-off of 10−25). A. Graphical representation (Sequence Logo, [48]) of 895 aligned sequences of the conserved Motif 2 identified in K. veneficum plastid sequences. B. 895 aligned DNA sequences containing Motif 2 and trnE in putative minicircles shown in MacClade (pretty print matrix). Left panel; forward and complementary reads aligned together. Right panel; forward reads and complementary reverse reads aligned in separate blocks. (TIFF) [file pone.0038809.s001.tiff]

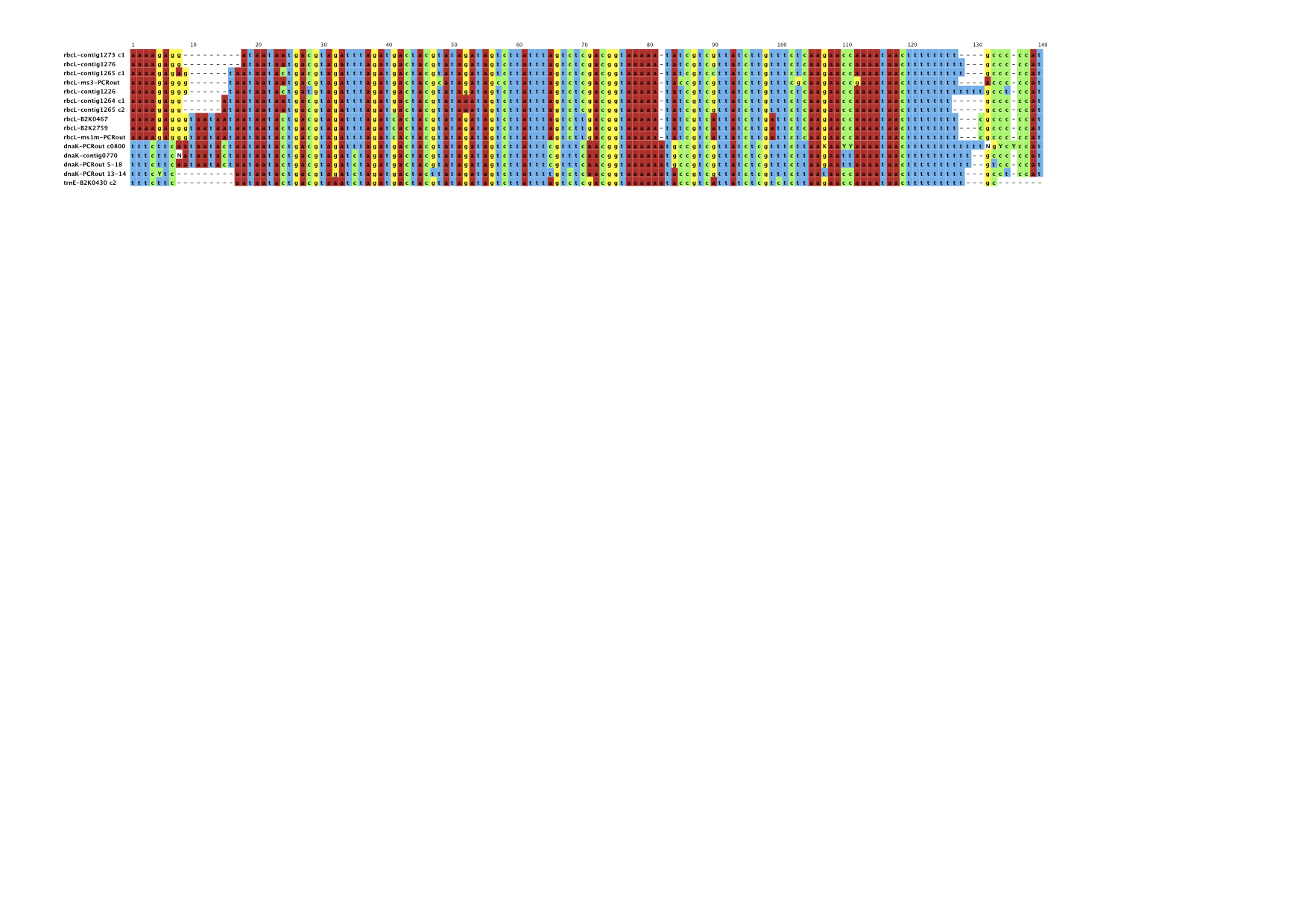

Supplement: Figure S2 — Aligned sequences of Motif 2 in library clones. The gene structures of the library clones are shown in Figures 2 and 4. (TIFF) [file pone.0038809.s002.tiff]

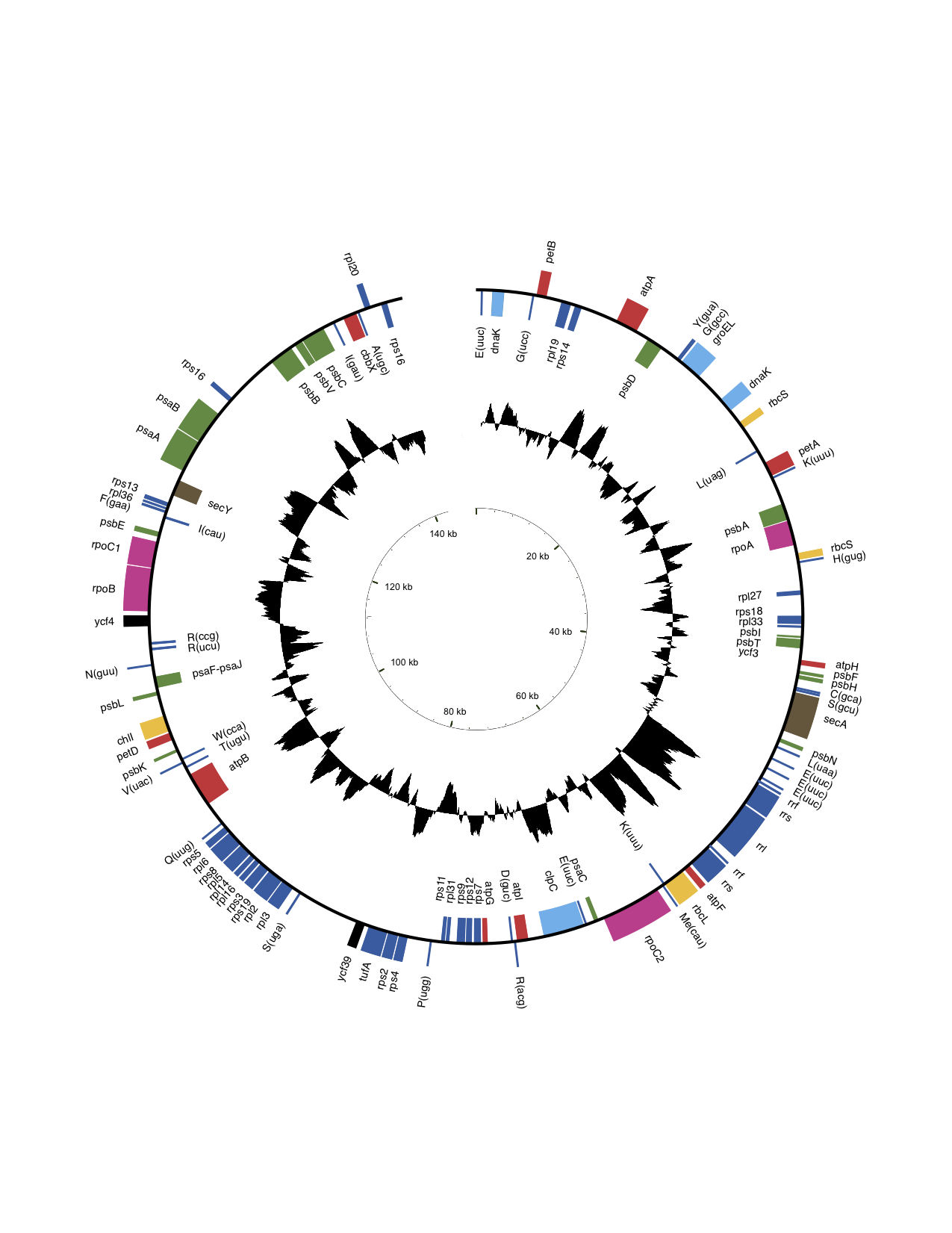

Supplement: Figure S3 — GCView [49] of the conventional plastid genome [21] of Karlodinium veneficum . (TIFF) [file pone.0038809.s003.tiff]

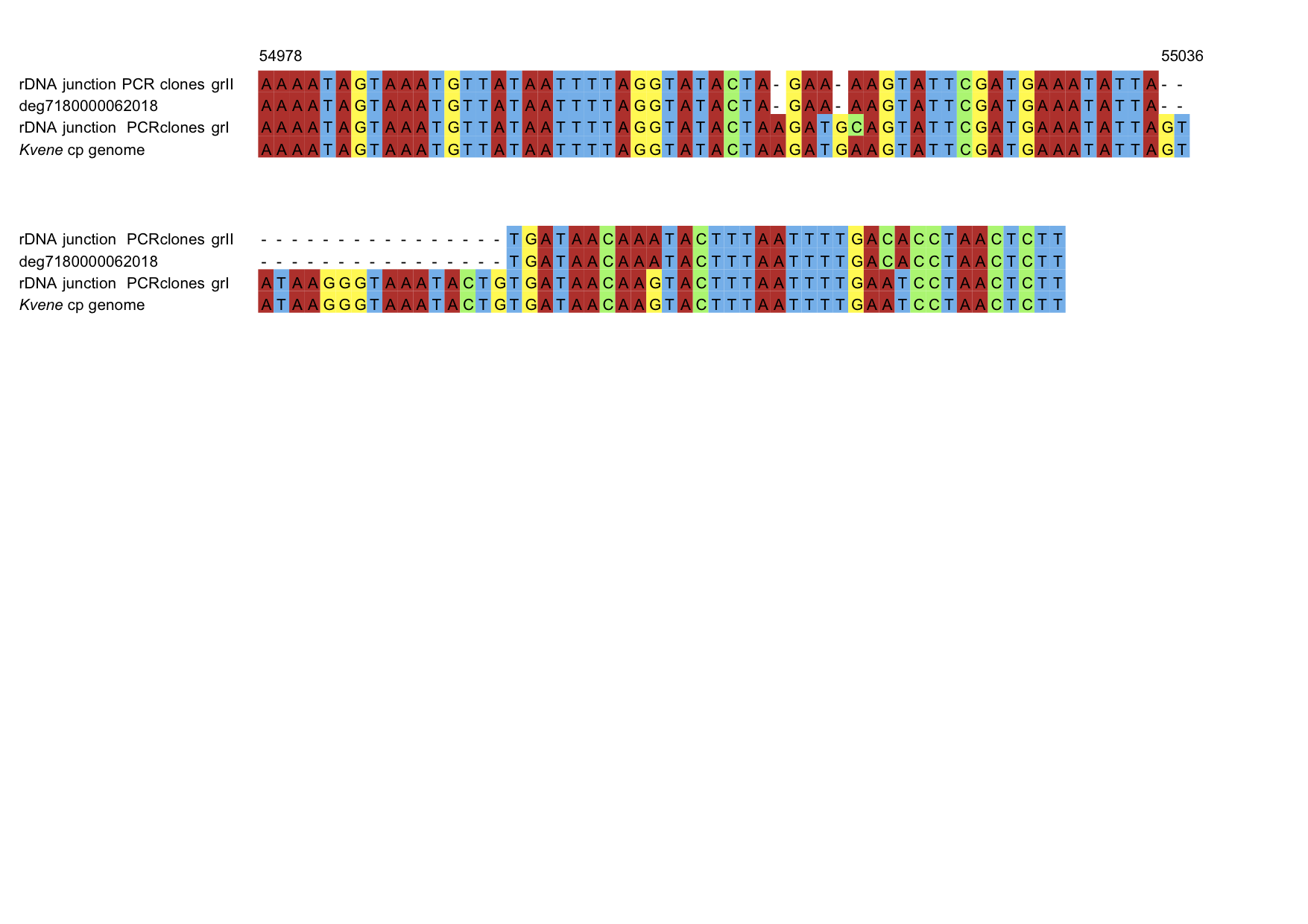

Supplement: Figure S4 — Aligned sequence connecting units of plastid ribosomal genes. The location the sequence in the plastid genome of K. veneficum is indicated above the sequences. (TIFF) [file pone.0038809.s004.tiff]
