# Supplementary material for: Genome Fragmentation Is Not Confined to the Peridinin Plastid in Dinoflagellates
Source: PLoS One. 2012 Jun 18;7(6):e38809. doi: 10.1371/journal.pone.0038809 (PMC3377699; doi:10.1371/journal.pone.0038809)
Supplement: Table S1 — PCR primers used for outwards directed amplification of putative minicircle genes. (DOCX) [file pone.0038809.s005.docx]

| Primer name | Primer sequence | PCR product size |
| --- | --- | --- |
| dnaK_1937out  dnaK_1079out | 5-CTTGGCGTTGTGAATCATTAAAA  5-ATCTTCATATTCTGCAAGGGGA | Ca. 2000 bp |
| KrbcL_FLout  KrbcL_RLout | 5-GATGCAGTTAGGTTTGCTAGTGA  5-CGTGCTGGTAATTCGGCGTATGC | Ca. 3000 bp  ”ms 3” |
| KrbcL_FLout  KrbcL_Rout | 5-GATGCAGTTAGGTTTGCTAGTGA  5-AATGTTACTGGGGCAACCATGG | Ca. 1800 ”ms 1” |
