# Supplementary material for: Genome Fragmentation Is Not Confined to the Peridinin Plastid in Dinoflagellates
Source: PLoS One. 2012 Jun 18;7(6):e38809. doi: 10.1371/journal.pone.0038809 (PMC3377699; doi:10.1371/journal.pone.0038809)
Supplement: Table S2 — PCR primers used for amplification of probes used in Southern blot hybridization. (DOCX) [file pone.0038809.s006.docx]

| Probe name | Primer name | Primer sequence | PCR product size |
| --- | --- | --- | --- |
| rbcL – probe 1 | KrbcLf  KrbcLr | 5-TCACTAGCAAACCTAACTGCATC  5-GCATACGCCGAATTACCAGCACG | 564 bp |
| rbcL – probe 2 | KrbcL-pf  KrbcL-pr | 5-AAAACTTGAGGGCGATCCTT  5-GAAACTAACCCCATGCCAGA | 403 bp |
| rbcL – probe 3 | 3-ms3  5-ms3 | 5-CCCTTTCACGGAAAAAGCTA  5-CTTATTACAAATACCCTGCCAAG | 522 bp |
| rbcL – probe 4 | 1-ms3  2-ms3 | 5-CGCAAAGAGTACCACAGCAA  5-CGTCAAAGGGAGCTGTCCTA | 1001 bp |
| dnaK | dnaK_1937  dnaK_1079 | 5-TTTTAATGATTCACAACGCCAAG  5-TCCCCTTGCAGAATATGAAGAT | 882 bp |
| LSU | C3_2667L  C3_4492in | 5-CGAGCGAACGCGGGAAAACA  5-TTCCGACCCGCACGAATGGT | 1844 bp |
