# Supplementary material for: Genome Fragmentation Is Not Confined to the Peridinin Plastid in Dinoflagellates
Source: PLoS One. 2012 Jun 18;7(6):e38809. doi: 10.1371/journal.pone.0038809 (PMC3377699; doi:10.1371/journal.pone.0038809)
Supplement: Table S3 — Sequence coverage in 454 sequences generated from pooled CsCl fractions 11–14. The coverage is calculated as the read depth for each base, averaged over the contig. (DOCX) [file pone.0038809.s007.docx]

| Large contigs*) | Coverage |
| --- | --- |
| Cp rDNA | 410-451 |
| Nuclear rDNA | 64-146 |
| Mt DNA | 57 and lower |
| Other cp DNA**) | 17 and lower |

*) Large contigs in Newbler assembly; 500 bp and more

**) Other cp DNA: Sequences with hits to the conventional plastid genome
